# Emerging Non-Pharmacological Approaches in Endometriosis: Mechanistic Insights into Phototherapy, Hyperthermia, and Acupuncture—Literature Review

**DOI:** 10.3390/jcm15114136

**Published:** 2026-05-27

**Authors:** Iga Szukalska, Maciej Ziętek, Edyta Zagrodnik, Małgorzata Szczuko

**Affiliations:** 1 Department of Bromatology and Nutritional Diagnostics, Pomeranian Medical University in Szczecin, 71-460 Szczecin, Poland; malgorzata.szczuko@pum.edu.pl; 2 Department of General Pharmacology and Pharmacoeconomics, Pomeranian Medical University in Szczecin, 71-460 Szczecin, Poland; maciej.zietek@pum.edu.pl; 3 Clinical Department of Anesthesiology and Intensive Care of Adults and Children, Pomeranian Medical University in Szczecin, 71-460 Szczecin, Poland; edyta.zagrodnik@pum.edu.pl

**Keywords:** endometriosis, phototherapy, heating, acupuncture, pain

## Abstract

**Background/Objectives**: Endometriosis is a chronic condition affecting women of reproductive age. Its symptoms have a negative impact on the quality of life and fertility of many women in the population. The aim of this literature review was to examine the use of phototherapy, heating and acupuncture in the treatment of endometriosis. **Methods**: A structured review of the available literature using the PubMed, Embase and Scopus databases, including studies from the last 10 years and with full free access, was applied. The literature search was conducted using the keywords: “endometriosis”, “phototherapy”, “heating” and “acupuncture”. **Results**: Phototherapy, including photothermal (PTT) and photodynamic therapy (PDT), demonstrated promising results in preclinical animal models, suggesting a potential for reducing endometrial lesions, primarily through mechanisms involving apoptosis, necrosis, and oxidative stress. However, most studies were limited to animal models. Thermal interventions, including magnetic hyperthermia and perioperative heating strategies, were associated with pain reduction, although improper use may lead to adverse effects such as erythema. Acupuncture showed effectiveness in reducing pain and improving quality of life, although its effects may be temporary and supported mainly by small-scale studies and case reports. **Conclusions**: Studies available in the literature demonstrate the effectiveness of the phototherapy effect, utilizing the mechanism of apoptosis or necrosis, in eliminating endometrial tissue and reducing pain. Acupuncture, derived from Traditional Chinese Medicine, also reduces pain. Non-pharmacological interventions may provide supportive benefits in the management of endometriosis, particularly in pain reduction and lesion control.

## 1. Introduction

Endometriosis is a chronic inflammatory gynecological condition with a systemic nature [1]. Epidemiological data published by the World Health Organization indicates that endometriosis affects approximately 10% of reproductive-age women globally, representing roughly 190 million individuals worldwide [2]. Despite there being so many affected women, endometriosis poses a clinical challenge, as diagnosis takes an average of seven to ten years, and the actual number of cases may be higher [3]. The essence of the disease lies in the presence of tissue structures resembling the endometrium but occurring outside the endometrium and the uterine muscle. They may be found in the intestines, bladder, lymph nodes and in rarer cases even in the lungs, diaphragm, or pericardium. Their presence is accompanied by chronic inflammation [1,4]. Inflammation plays a significant role in the development of pain and infertility associated with endometriosis. It contributes to the formation of lesions, but also to the occurrence of symptoms. Inflammatory mediators stimulate peripheral pain receptors. This results in chronic pelvic pain and long-term persistent inflammation within the abdominal cavity, which negatively affects fertility and patients’ quality of life. It impairs the multi-stage development of ovarian follicles and compromises gamete function and fertilization [5]. The mechanism of infertility in people with endometriosis is complex, as it is associated with anatomical abnormalities, including adhesions and fibrosis. Endocrine and immune disorders, as well as oxidative stress, are also present. It is therefore estimated that infertility affects around 30–50% of patients with endometriosis [6].

### 1.1. Etiology

The development of endometriosis is influenced by a number of factors. Genetic, immunological and hormonal factors contribute to its onset [3]. Hypoxia plays a significant role in the development of endometriosis, and DDR2 is a key gene associated with hypoxia in endometriosis and a promising diagnostic and therapeutic biomarker [7]. The pathogenesis of endometriosis has not been definitively clarified yet. The two main pathogenic theories are the retrograde menstruation (RM)/implantation theory and the embryonic remnant/coelomic metaplasia theory, based on the development of endometrial tissue at ectopic sites [8]. RM can be considered a factor initiating the development of endometrial lesions, while not excluding the involvement of both hereditary and tissue-specific genetic and epigenetic modifications as factors contributing to the development of the disease [8]. Endometrial implants commonly occur on the ovaries and peritoneum but can spread to distant sites such as the lungs [9]. Another of the main factors is retrograde menstrual flow, although its occurrence is not synonymous with the presence of endometriosis. Severe oxidative stress and inflammation play a key role in the pathophysiology of endometriosis. Patients with endometriosis have higher homocysteine levels, which correlates with hormonal disturbances. Higher NK-κB is observed in these patients, which exacerbates inflammation and the production of inflammatory mediators: TNF-α and the pro-inflammatory interleukins IL-6 and IL-8. This results in an intensification of the symptoms experienced [10].

### 1.2. Symptoms

The most reported problems are pelvic pain and painful periods, sexual intercourse, bowel movements, and urination [11], as well as symptoms of fatigue, depression, and irritable bowel syndrome (IBS). Symptoms of IBS may include non-specific abdominal discomfort, as well as episodes of diarrhea and/or constipation. In many patients, symptoms are exacerbated or compounded by co-existing conditions [1]. The symptom of secondary dysmenorrhea leads most women to a diagnosis of endometriosis. The pathophysiology of dysmenorrhea is complex, comprising factors such as immunological disorders, increased prostaglandin production, adhesions within the pelvis, or neurological factors [12].

Women affected by this condition are at greater risk of developing mental health problems or sexual dysfunction caused by the pain they experience [13]. Patients report symptoms of anxiety and depression. The constant pain they experience disrupts their well-being and social relationships, which also contributes to difficulties in adhering to medical advice [14]. From a public health perspective, the World Health Organization classifies endometriosis as a major socio-economic burden, driven by substantial direct healthcare costs and massive indirect costs related to loss of workplace productivity and disrupted education [2].

Clinically, endometriosis can be classified as

- Superficial: small, flat, shallow lesions present in the lesser pelvis;
- Ovarian: cysts or endometrial tissue present in the ovaries;
- Deep: invading 5 mm or more beneath the peritoneum [1].

### 1.3. Treatment

One of the standard forms of treatment is pharmacotherapy. The classes of drugs used include analgesics, nonsteroidal anti-inflammatory drugs (NSAIDs), combined oral contraceptives, and gonadotropin-releasing hormone (GnRH) agonists [15]. Other hormonal therapies are also used. One of these is an intrauterine device that releases levonorgestrel, a progestogen (dienogest), and reduces estrogen production [1]. Although pharmacological treatment alleviates pain, it causes a range of side effects and adverse reactions. Hormonal therapies may lead to symptoms of hypoestrogenism. Consequently, this can result in reduced bone mineral density, mood disorders, or drug dependence [14]. Furthermore, women undergoing hormone replacement therapy are at a higher risk of developing certain cancers but also face a greater risk of cardiovascular disease [12]. Danazol, used in treatment, may be associated with androgenic symptoms, including acne and weight gain. It also plays a role in increasing LDL cholesterol levels and has a potential link to occurrence of ovarian cancer. Treatment with progestogens causes bloating, acne and spotting. The lipid profile is also disrupted in the HDL fraction. This increases the risk of cardiovascular complications [16]. Gonadotropin-releasing hormone agonists cause side effects in the form of hypoestrogenic states, like the symptoms of the menopause, as well as vaginal atrophy [17]. Consequently, although these drugs offer therapeutic benefits, a significant proportion of women discontinue pharmacotherapy due to these side effects [18].

Laparoscopy is frequently used in surgical treatment, as it allows for the removal of lesions. It is performed using a specialized robot, both for treatment and for advanced diagnostic purposes [1]. Surgical treatments also include ablation of the uterosacral nerves, as well as radical hysterectomy in certain cases [10]. Methods of combined surgical–pharmacotherapeutic treatment or assisted reproductive technologies are also used [17]. Surgical interventions, particularly laparoscopic procedures, help to reduce pain and improve quality of life. However, the effects of surgical intervention are not permanent in all patients. This is influenced by various factors, such as the stage of endometriosis, the surgical technique used, and post-operative care [3]. The main factor causing the non-permanent effect of surgery is the presence of small residual endometriosis lesions in the surgical field [19].

Several non-pharmacological approaches have been investigated for endometriosis-associated pain, including physiotherapy, exercise interventions, psychological therapies, dietary modifications. However, the scope of the present review was intentionally focused on selected emerging non-pharmacological modalities with distinct mechanistic profiles—phototherapy, thermal interventions, and acupuncture.

## 2. Materials and Methods

**Figure 1:**
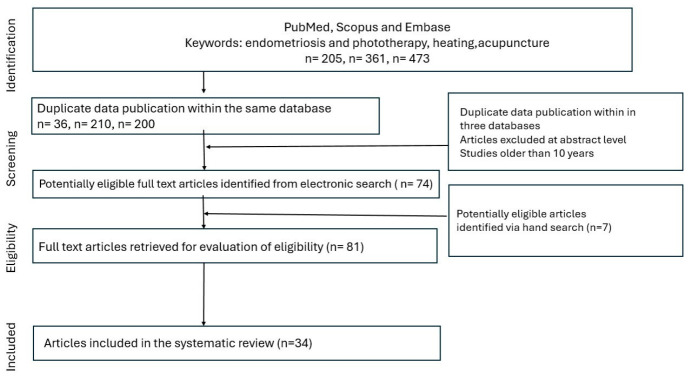
PRISMA flow diagram of study identification, screening, eligibility assessment, and inclusion in the structured review.

In a study on complementary treatments for endometriosis, a systematic literature review was conducted with the aim of compiling and analyzing available research on the subject, using PRISMA guidelines. The inspection was not registered. The structured review was carried out using the PubMed, EMBASE and SCOPUS databases. The following keywords and Medical Subject Headings (MeSH) terms were used: “endometriosis” AND (“phototherapy” OR “photothermal therapy” OR “photodynamic therapy” OR “hyperthermia” OR “heating” OR “acupuncture”). Inclusion criteria: studies involving patients or animal models with endometriosis; studies evaluating phototherapy, thermal therapy, or acupuncture; original research articles (RCTs, observational studies, case reports); English language; full-text availability. Exclusion criteria: review articles, conference abstracts, studies lacking relevant outcomes. The following restrictions were applied in the review of the available literature: studies from the last 10 years, full free access, and relevance to the search terms. Searches were limited to English-language full-text articles. Records were independently screened by at least two reviewers at successive stages of selection. Discrepancies were resolved through discussion and consensus or by involving a third reviewer. After screening and full-text assessment, studies meeting the inclusion criteria and deemed methodologically sound were retained for qualitative synthesis and discussion. Due to heterogeneity and inclusion of case reports and animal studies, a formal meta-analysis was not performed. The methodological quality of the included studies was assessed qualitatively according to study design, sample size, methodological transparency, and risk of bias considerations, in line with PRISMA recommendations (check list attached Table S1). Given the high heterogeneity and the inclusion of both animal models and case reports, a formal scoring system was not applied, but each study was evaluated for internal validity and clinical relevance. Due to substantial heterogeneity, no formal pooled scoring or meta-analysis was performed (Figure 1).

## 3. Results

### 3.1. Phototherapy

Phototherapy is an emerging area of research in the treatment of endometriosis. In the treatment of endometriosis, the efficacy of phototherapy is primarily attributed to two mechanisms. One of these is photothermal therapy (PTT), which acts via laser-induced hyperthermia while causing only minimal damage to surrounding tissues [20]. In experimental settings, photothermal therapy has shown potential as a promising experimental approach with few side effects. It involves the selective destruction of diseased cells by generating heat using photoactive, non-toxic agents. Research was not conducted on humans, but in the tested mice, a single PTT procedure already causes a reduction in transplanted endometrial lesions. In addition, no adverse effects were observed in mice subjected to the procedure [19]. Photosensitive photothermal agents (PTA) have the ability to convert near-infrared (NIR) light into thermal energy, including hyperthermia. This leads to the destruction of abnormal cells or bacteria. This is caused by the denaturation of their proteins and the induction of cell death, which occurs as a result of apoptosis triggered by oxidative stress or mitochondrial damage [21]. Photodynamic therapy (PDT) leads to the formation of reactive oxygen species (ROS). The resulting reactive oxygen species contribute to cell destruction through circulatory disturbances and oxidative damage to biomolecules [20]. In a case report, photodynamic therapy (PDT) utilized a mechanism of destruction of the vascular endothelium, leading to their occlusion, which had a positive effect on reducing the vascularization of pathological lesions. The use of PDT in endometriosis localized in the lung eliminated hemoptysis after a single treatment. The photosensitizing agent (50 mg of hematoporphyrin) is administered intravenously approximately 24 h prior to laser exposure. Laser delivery is achieved internally; for instance, in pulmonary cases, a semiconductor laser (630 nm, 2 W) is delivered via a bronchoscope directly to the target segments. Importantly, no skin photosensitivity was noted during the follow-up period [22]. An advantage of PDT is the low incidence of adverse effects and high patient tolerance. Furthermore, it provides treatment targeted at limited and superficially localized pathological lesions. Photodynamic therapy may be helpful when a patient cannot tolerate pharmacological treatment or when such treatment is ineffective [22]. In studies to date, the combination of PTT and PDT has been shown to mitigate the adverse effects caused by these therapies [20]. Furthermore, PTT and PDT therapies have a localized effect, as no damage to surrounding structures is observed [19,22].

Zhong Q et al. [20] applied photothermal therapy combined with photodynamic therapy using NIR (808 nm) in an animal model, specifically in mice. The aim of this study was to develop modern approaches to the treatment and diagnosis of lesions associated with endometriosis. Furthermore, the aim was to apply a method that effectively reduces side effects. In the study, a molecular probe consisting of cRGD-ILD and ILD was developed; when combined with PTT/PDT under NIR irradiation, this produced a thermal effect and raised the temperature to 42 °C, resulting in thermal ablation and increased intracellular ROS production. The use of this combined therapy demonstrates new possibilities for imaging, monitoring treatment and undertaking interventions [20]. Moses A S et al. [19] also conducted their research on mice. In their study, they used PTT and near-infrared light (700–900 nm) to visualize endometrial lesions transplanted into mice using fluorescence. The result of this study was the reduction in experimentally induced lesions in animal models in the mice after just the first treatment. Furthermore, no adverse effects were observed. The SiNc-NP nanoparticles used enabled the localization of the lesions and their effective destruction using the thermal effect [19]. Wang T et al. [22] presented a case report of a 34-year-old woman with pulmonary endometriosis (PEM) and adenomyosis. The patient experienced hemoptysis on the first and second days of her menstrual period. The patient reported previous attempts at treatment with antibiotics and GnRH antagonists. During her hospitalization, experimental treatment using PDT was administered. This resulted in the cessation of hemoptysis after the first treatment and complete resolution of the lesion in the left upper lobe of the lung. No adverse effects were observed in the patient. PDT may represent a potential option in highly selected cases; however, current evidence is limited to isolated case reporting. Especially in patients experiencing hemoptysis in the context of pulmonary endometriosis, however, this requires further research [22]. The studies described are presented below in Table 1.

### 3.2. Heat Therapy

Magnetic hyperthermia has not previously been investigated as a potential treatment for endometriosis. This therapy uses magnetic nanoparticles, which, when exposed to an alternating magnetic field (AMF), are capable of generating heat. This allows for a localized increase in temperature without damaging surrounding tissues. Depending on the temperature generated, different effects are observed. Temperatures in the range of 42–46 °C can cause cell apoptosis, whilst temperatures above 46 °C can cause thermal ablation. An alternating magnetic field induces hyperthermia above 42 °C, even in deep-seated tissues, without causing adverse effects on physiological processes in humans or animals [23]. Park Y et al. [23] conducted animal studies using magnetic hyperthermia. They used an alternating magnetic field (AMF) to raise the temperature of iron oxide nanoparticles. In the cited preclinical study, iron oxide nanoparticles were incorporated into nanocarriers functionalized with ligands targeting vascular endothelial growth factor receptor-2 (VEGFR-2/KDR), a receptor associated with angiogenic endometriotic lesions. This design aimed to enhance preferential accumulation within endometriotic tissue compared with surrounding healthy structures. Upon exposure to an alternating magnetic field, localized heat generation induced cytotoxic hyperthermia within nanoparticle-enriched lesions. However, this selectivity remains relative rather than absolute, and its safety and reproducibility in humans have not yet been established. The result of the study was the elimination of transplanted endometrial lesions in mice after a single treatment using the magnetic hyperthermia effect [23]. The AMF variable magnetic field effectively raises the temperature in the tissue to above 50 °C following the administration of hexagonal iron oxide nanoparticles, which may destroy endometriotic tissue, which may offer preliminary evidence for future treatment options; however, human safety data and clinical trials are required to confirm its translatability. In the cited experimental model, localization of magnetic nanoparticles was achieved through direct administration into lesion-bearing experimental tissue rather than validated selective targeting in human disease. Therefore, the feasibility of selective tissue targeting in clinical practice remains uncertain.

### 3.3. Heat Therapy for the Treatment of Pain

Intraoperative management may influence the level of pain experienced following laparoscopic surgery in patients with endometriosis. Positive effects have been reported following the use of humidified, warm insufflation gas during surgery and a heated blanket with warm air circulation [24]. Patients with endometriosis use thermotherapy, in the form of heat packs, to reduce chronic pelvic pain and alleviate dysmenorrhea. However, this method carries a risk of developing erythema ab igne (EAI), a brown, reticulated skin pigmentation. These lesions appear as a result of skin exposure to heat below the burn threshold. EAI is not merely a cosmetic defect but has the potential to become malignant. As a result of this condition, squamous cell carcinoma, Merkel cell carcinoma or basal cell carcinoma may develop [25].

The study by Breuer M et al. [24] described the effects of using moistened, warm insufflation gas during laparoscopic surgery and the use of a heating blanket. Among 150 women with endometriosis undergoing laparoscopy, a group of 48 women was formed in whom this method was applied. The result of this modification was a reduction in pain symptoms assessed by the patients using the VAS scale. A reduced use of ibuprofen was also noted in this group on the second day after surgery. This study shows that the intraoperative use of warm, moist insufflation gas may be beneficial in patients with endometriosis in reducing the pain they experience [24]. Scurtu F et al. [25] presented a case report of a 33-year-old woman suffering from deep endometriosis. Due to the pain she was experiencing, the patient used heating pads for too long, which caused skin changes in the form of EAI. This patient’s case highlights the need to develop effective forms of pain management for patients with endometriosis and the need for education on safe ways of managing pain [25]. The studies described are presented below in Table 2.

In studies conducted on rats, an intervention involving the administration of the gonadotropin-releasing hormone (GnRH) agonist was carried out. The administration of GnRH caused an increase in body temperature in the rats studied, which may be similar to the hot flushes characteristic of menopausal symptoms. An intervention was carried out involving the administration of estradiol valerate (E2) tablets and ultrasonically processed Idris tridentata (ICR) preparations to create a homogenous suspension dissolved in saline solution. The results showed that the use of E2 and ICR led to a more rapid decrease in temperature. At the same time, the use of ICR did not significantly affect sex hormone levels in the rats studied. Furthermore, changes occurring in the ovaries were assessed. Histopathological studies showed that the use of GnRH leads to a reduction in the number of growing and mature follicles [26]. The use of ICR does not affect serum sex hormone levels, which may make it a safe treatment option. The use of E2 and ICR effectively alleviates symptoms such as weight gain and fever.

### 3.4. Acupuncture

Acupuncture, which forms part of Traditional Chinese Medicine, is used to treat illnesses, but is also a common method of pain relief in Traditional Chinese Medicine. The acupuncture evidence base was dominated by case reports and heterogeneous multimodal interventions, substantially limiting comparative interpretation. The mechanism of its analgesic action is based on the Traditional Chinese Medical theory of the zang-fu organs, which describes the functioning of the human body and its connections via a network of meridians. According to Traditional Chinese Medicine, pain in endometriosis results from a stagnation of blood or Qi energy within the uterus. Stimulation of specific acupuncture points improves the circulation of blood and Qi via the nervous system, thereby reducing pain [27]. Acupuncture is also used as part of traditional Korean medicine. This therapy includes herbal medicines, acupuncture, moxibustion and fumigation therapy. Moxibustion involves the application of heat to areas associated with the condition. Its use results in a reduction in the levels of prostaglandins and histamine in the blood. Fumigation therapy is a ritual involving the exposure of the external genitalia to steam enriched with herbs [28]. The use of acupuncture in clinical practice is employed to reduce pain, regulate the menstrual cycle and reduce the size of lesions associated with endometriosis [29]. The mechanism of action of acupuncture is multifaceted. These treatments modulate the immune response and support liver function. This action stimulates the nervous system to release neurotransmitters and hormones. Their release reduces stress and improves the circulation of blood and energy within the body [30].

The study by Li P S et al. [31] recruited 106 women with endometriosis who were experiencing pain. The patients were divided into a group receiving acupuncture and a control group. The intervention lasted for 12 weeks. After this period, a reduction in pain, assessed using the VAS scale, was observed in the group receiving acupuncture. However, after a longer period of 24 weeks, no statistically significant differences were observed between the groups participating in the study. This study demonstrated the efficacy of the acupuncture used but also revealed its temporary nature [31]. Ai K, L et al. [32] observed the effects of acupuncture therapy among 27 patients with ovarian endometriosis. The patients were recruited from a group of 81 women undergoing in vitro fertilization (IVF-ET). The 27 women in this group were treated with a GnRH antagonist and acupuncture. In this group, a reduction in pain symptoms during the perimenstrual period was observed. A higher embryo quality index was also noted in this group [32]. Payne J. A. et al. [33] presented a case report of a 43-year-old woman with endometriosis. The patient underwent acupuncture in combination with herbal medicine. Following this combined therapy, a reduction in pain and an improvement in the patient’s general well-being were observed over the course of a 6-month treatment period [33]. Martin B. R [10] presented the case of a 36-year-old woman with endometriosis. The intervention consisted of acupuncture combined with supplementation with 400 mg of magnesium citrate, B-100 complex, 1000 mg of turmeric, 1000 mg of bromelain, 141 mg of calcium carbonate and 540 mg of Echinacea purpurea. The result of the acupuncture sessions was a reduction in the pain experienced by the patient, rated on a scale of 1–10. Additionally, the patient’s accompanying migraines and tension headaches subsided. The intervention described, involving the use of acupuncture and supplements, alleviated the pain associated with endometriosis. The positive outcome of the treatment may have been the result of a synergy that has been observed in previous human studies [10]. Kim H. W. et al. [28] also presented a case report of a 32-year-old woman with recurrent endometriosis. She was treated with a combined therapy of traditional Korean medicine, comprising herbal formulas combined with acupuncture, moxibustion and fumigation therapy. The result of this treatment was the elimination of endometriosis recurrences and a reduction in pain, as well as the elimination of blood clots. This study shows that the use of traditional Korean medicine may be a way of helping patients with endometriosis [28]. The study by Du X et al. [29] presents a case report of a 38-year-old patient with a recurrence of a painful mass. The patient had a history of endometriosis surgery. She underwent acupuncture treatment combined with mass-dissolving therapy. The result of these interventions was the complete elimination of the pain she was experiencing; furthermore, a reduction in the size of the lesion was observed. The patient also reported a general improvement in the quality of her sleep and her mood [29]. Zhu J et al. [30] described the case of a 29-year-old female patient with primary infertility and endometriosis localized in the left ovary. The patient underwent acupuncture as she did not consent to other treatment methods. The outcome of the intervention was a reduction in the size of the endometrial cyst on the left ovary and a spontaneous pregnancy with a confirmed embryo [30]. The studies described in this section are presented below in Table 3.

## 4. Discussion

Endometriosis is a fairly common condition in the general population, but it requires particular attention as it affects many women of reproductive age [33]. Its symptoms significantly reduce patients’ quality of life, affect mental health, and the persistent chronic pain impacts overall well-being [13]. Endometriosis can also occur outside the uterus. In women of reproductive age, it presents as a cyst within the ovary and is referred to as ovarian endometriosis. Its presence in this location is often associated with infertility [30].

The results of the literature review show that various methods to support the treatment of endometriosis and its associated symptoms are described in the literature. It is essential to expand our knowledge of alternative treatment methods, as many patients experience side effects associated with pharmacotherapy and recurrences following surgical treatment. An analysis of the studies and case reports presented indicates that phototherapy, heating and acupuncture have a positive effect in the treatment of endometriosis. The use of modern phototherapy, primarily photothermal therapy (PTT) and photodynamic therapy (PDT), as described in the studies, shows promising results in eliminating endometriosis lesions. Furthermore, no adverse effects of the methods used were reported [20]. A limitation of studies utilizing photothermal effects is that these therapies have been applied to animal models, particularly mice with transplanted endometriosis.

Another method analyzed in the literature is the thermal method. The use of magnetic hyperthermia also effectively eliminates endometrial tissue after a single application. In this therapy, a temperature of 42–46 °C induces apoptosis, while a temperature above 46 °C causes coagulative necrosis or both, depending on exposure time and exact temperature. Specifically, recent advancements in nanotechnology have led to the development of new generations of KDR-MN with significantly enhanced heating efficiency [23]. Heat can be utilized in many ways to reduce the pain associated with endometriosis. Another promising area of research involves the use of warm, moist insufflation gas during laparoscopic treatment. A reduction in pain was observed among patients following endometriosis surgery [24]. The improper use of heat to relieve pain can have adverse effects. It is therefore important to remember that it must be used in accordance with safety guidelines. An example of a patient who overused heat to alleviate pain is described in an article by Scurtu F et al. [25]. The final method analyzed to support the treatment of endometriosis is acupuncture. This method is most frequently cited in recent studies and is effective in reducing pain. This method is often used in combination with interventions incorporating traditional Chinese or Korean medicine. In addition to reducing pain, acupuncture appears to be effective in treating infertility in women with endometriosis. In the study by Ai K L et al. [32], in addition to alleviating pain, an improvement was observed in parameters relevant to the in vitro fertilization procedure [32]. Meanwhile, the results of Zhu J et al. [30] regarding a case report also indicated the potential use of acupuncture in the treatment of infertility. Following acupuncture treatment, there was a reduction in endometriosis lesions, and the patient became pregnant [30]. Despite the promising results of the studies reviewed, it is important to highlight their significant limitations. The limitations of the studies presented were that modern photothermal therapies were conducted mainly on animal groups, particularly mice. Only one study presented a case report of a female patient. Furthermore, in the other methods, the study groups are small or consist of single-case reports. Consequently, it is not possible to generalize the findings to the population of women suffering from endometriosis.

**Figure 2:**
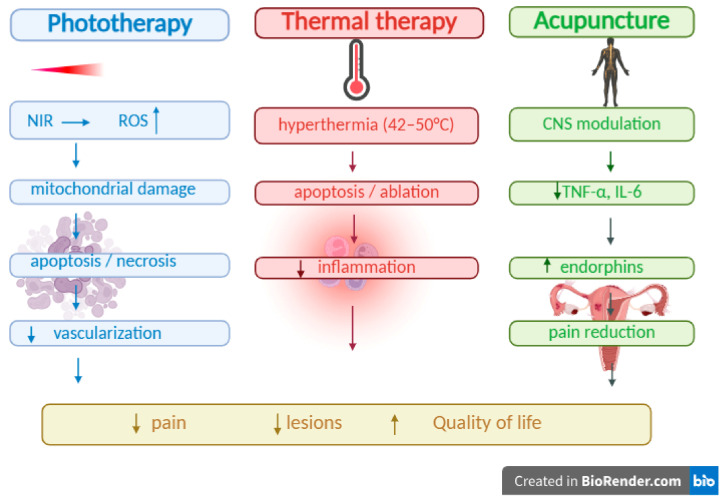
Proposed mechanisms potentially involved in the observed effects of thermal therapy and acupuncture in endometriosis. NIR—Near-Infrared Radiation; ROS—Reactive Oxygen Species; TNF—Tumor Necrosis Factor; IL-6—Interleukin-6; Created in Bio-Render. Szukalska/Szczuko. 10 May 2026 https://www.biorender.com.

Phototherapy, including photothermal (PTT) and photodynamic therapy (PDT), induces apoptosis and necrosis of endometriotic lesions through hyperthermia and reactive oxygen species (ROS) generation. Thermal therapy, such as magnetic hyperthermia, leads to localized tissue ablation and modulation of inflammatory pathways. Acupuncture exerts analgesic and regulatory effects via modulation of the nervous system, reduction in pro-inflammatory cytokines (e.g., TNF-α, IL-6), and improvement of blood circulation [8]. All approaches may contribute to pain reduction, decreased lesion size, and improved quality of life in patients with endometriosis. The proposed mechanisms are summarized in Figure 2.

## 5. Limitations of the Study

In several cited clinical reports, acupuncture or other non-pharmacological interventions were applied alongside additional therapeutic approaches, such as herbal medicine, supplementation, GnRH therapy, or multimodal supportive care. This co-intervention design makes it impossible to attribute observed clinical improvements exclusively to acupuncture itself and substantially limits causal interpretation.

## 6. Conclusions

The analyzed non-pharmacological interventions, including phototherapy, thermal therapy, and acupuncture demonstrate potential as supportive strategies in the management of endometriosis. Phototherapy appears particularly promising due to its targeted mechanism of action involving ROS generation and selective tissue ablation. Thermal interventions may effectively reduce pain, although safety considerations are crucial. Acupuncture provides symptomatic relief, particularly in pain management, but its long-term efficacy remains uncertain.

Despite these promising findings, the available evidence is limited by methodological heterogeneity, small sample sizes, and a predominance of animal studies and case reports. Therefore, these approaches should currently be considered complementary rather than primary treatment strategies. Future research should focus on large-scale randomized controlled trials and mechanistic studies to better define their clinical role and optimize treatment protocols.

## Figures and Tables

**Table 1:** Summary of Phototherapy-Based Interventions in Endometriosis, highlighting mechanisms (PTT/PDT) and outcomes in animal models and clinical case reports.

Author	Method	Study Group	Results	Conclusions
Zhong Q et al. [20]	PTT/PDT + NIR (808 nm)	Mice	The photothermal effect was achieved using a laser (808 nm), resulting in a temperature rise to 42 °C, which exceeds the cytotoxic threshold for the cell. Combined PTT and PDT contributes to thermal ablation and leads to the production of ROS.	The use of cRGD-ILD probes enables effective PTT/PDT using NIR, due to their accumulation in endometriotic lesions and EESC cells.
Moses AS et al. [19]	PTT + NIR (near-infrared 700–900 nm)	Mice	A single PTT treatment eliminated endometriosis implants without any apparent side effects	The SiNc-NP nanoparticles developed enable the diagnosis and treatment of endometriosis. They help to locate lesions and destroy them using the photothermal effect.
Wang T et al. [22]	PDT	A patient with pulmonary endometriosis (PEM)	A single course of PDT treatment stopped the hemoptysis and completely resolved the lesions in the left upper lobe of the lung, without any side effects	PTD therapy may be effective in treating hemoptysis in people with PEM.

PDT—Photodynamic Therapy; PTT—Photothermal Therapy; ROS—Reactive Oxygen Species; PEM—Pulmonary Endometriosis; NIR—Near-Infrared Radiation.

**Table 2:** Effects of Thermal Interventions on Pain and Endometrial Lesions.

Author	Method	Study Group	Results	Conclusion
Breuer M et al. [24]	Moist, warm insufflation gas + a heated blanket	Women (n = 48), total group (n = 150)	In the group treated with this method, there was a reduction in pain levels as assessed using the VAS scale, as well as lower ibuprofen consumption on the second day following the procedure.	The use of warm, humidified gas during laparoscopic surgery may help to reduce pain.
Scurtu F et al. [25]	Heating pads	Case report: a 33-year-old woman with deep endometriosis causing pain.	The patient developed EAI as a result of overusing heat to relieve pain; the procedure involved the excision of the endometrial lesions and subsequent pharmacological treatment of the skin lesions.	There is a need for effective pain management therapies for patients with endometriosis, in order to prevent the adverse effects of non-pharmacological treatments used by patients.

EAI—Erythema Ab Igne.

**Table 3:** Clinical and Case-Based Evidence on Acupuncture in Endometriosis.

Author	Method	Acupuncture Points	Study Group	Results	Conclusions
Li P S et al. [31]	Acupuncture or control group	CV4, on both sides: SP6, LR3, KI6, ST30	106 women with pain associated with endometriosis	At week 12 of treatment, the group that received acupuncture reported lower pain scores on the VAS scale; after 24 weeks, there were no statistically significant differences between the two groups	Acupuncture is an effective method for reducing pain, but its effects may be temporary.
Ai K, L et al. [32]	Acupoint application	CV8, on both side KI1 and Yashi	81 female patients undergoing in vitro fertilization (IVF-ET) 27 patients with ovarian endometriosis treated with GnRH and acupuncture 26 patients with ovarian endometriosis in the placebo group 28 patients with male-factor infertility	The treated group experienced a reduction in pain symptoms during the perimenstrual period, and the embryo quality index was also higher in this group. Acupuncture point therapy restores steroidogenic homeostasis	Therapy involving the application of herbal mixtures to acupuncture points alleviates pain and improves outcomes during in vitro fertilization
Payne J. A. [33]	Acupuncture and herbal medicine	CV3, 4, 5, ST 25, GV20, LI4, LR3, ST36, KI3, SP6, 9	A 43-year-old woman with endometriosis	A reduction in perceived pain and an overall improvement in well-being and physical and mental condition over the course of a 6-month treatment program	Combining acupuncture with herbal medicine helps to reduce pain, which may form part of the treatment for patients with endometriosis.
Martin B. R. [10]	Acupuncture + supplementation with magnesium citrate (400 mg), B-complex (100 mg), turmeric (1000 mg), bromelain (1000 mg), calcium carbonate (141 mg) and black cohosh (540 mg)	On both sides DU4, DU16, UB 20, UB23, One-sided KD3, KD6, SP3, SP6, GB 34 oraz 6 points in the lumbosacral region and the proximal part of the thigh	A 36-year-old woman with endometriosis	A reduction in pain to a level of 1/10, and the elimination of accompanying migraines and tension headaches	The acupuncture and supplements used help to relieve the pain.
Kim H, W et al. [28]	Herbal remedies + acupuncture + moxibustion + fumigation therapy	CV04, CV06, ST28, EX-CA1, SP06, ST36, SP09 and GV20	A 32-year-old woman with recurrent endometriosis	No recurrence, as confirmed by CT scan and CA-125 levels; resolution of pain symptoms; elimination of blood clots	A combined treatment using traditional Korean medicine may be helpful in treating recurrent endometriosis
Du X et al. [29]	Acupuncture with mass-releasing therapy	DU 20, DU24 On both sides DU26, ST25, CV4, SP15, SP6, SP9, SP5	A 38-year-old woman with a recurrence of a painful tumor, following previous surgery for endometriosis	Complete resolution (single case report) of pain, reduction in the lesion, and improvement in the patient’s sleep quality and mood	Acupuncture may help to alleviate pain and reduce the size of lesions associated with endometriosis. However, further research is needed
Zhu J et al. [30]	Acupuncture	Meridians of the pericardium, spleen, stomach, liver, Ren Mai, kidneys, large intestine, Du Mai and blanca	A 29-year-old female patient with primary infertility and endometriosis of the left ovary	A reduction in the size of the cyst on the left ovary and a single-embryo pregnancy.	Acupuncture, which forms part of Traditional Chinese Medicine, can reduce the size of cysts and help treat infertility

CT—Computed Tomography, GnRH—Gonadotropin-releasing Hormone, IVF-ET—In Vitro Fertilization and Embryo Transfer, VAS—Visual Analog Scale.

## Data Availability

The data can be found in the article.

## Supplementary Materials

The following supporting information can be downloaded at: https://www.mdpi.com/article/10.3390/jcm15114136/s1, Table S1: Checklist PRISMA [34].

## Abbreviations

The following abbreviations are used in this manuscript:

AMF	Alternating Magnetic Field
EAI	Erythema abigne
GnRH	Gonadotropin-Releasing Hormone
IBS	Irritable Bowel Syndrome
IL	Interleukin
NIR	Near-Infrared Radiation
NSAIDs	Non-Steroidal Anti-Inflammatory Drugs
PDT	Photodynamic Therapy
PTT	Photothermal Therapy
ROS	Reactive Oxygen Species
VAS	Visual Analog Scale
VEGF	Vascular Endothelial Growth Factor
